# Effect of Obesity Surgery on Taste

**DOI:** 10.3390/nu14040866

**Published:** 2022-02-18

**Authors:** Alhanouf S. Al-Alsheikh, Shahd Alabdulkader, Brett Johnson, Anthony P. Goldstone, Alexander Dimitri Miras

**Affiliations:** 1 Department of Metabolism, Digestion and Reproduction, Imperial College London, Hammersmith Hospital, London W12 0NN, UK; aa20@ic.ac.uk (A.S.A.-A.); shahd.alabdulkader@nhs.net (S.A.); brett.johnson@nhs.net (B.J.); a.miras@nhs.net (A.D.M.); 2 Department of Community Health Sciences, College of Applied Medical Sciences, King Saud University, Riyadh 11451, Saudi Arabia; 3 Department of Health Sciences, College of Health and Rehabilitation Sciences, Princess Nourah Bint Abdulrahman University, Riyadh 84428, Saudi Arabia; 4 PsychoNeuroEndocrinology Research Group, Division of Psychiatry, Department of Brain Sciences, Imperial College London, Hammersmith Hospital, London W12 0NN, UK

**Keywords:** bariatric surgery, gustation, taste perception, reward, appetitive, consummatory, sleeve gastrectomy, Roux-en-Y gastric bypass, adjustable gastric banding, sweet

## Abstract

Obesity surgery is a highly efficacious treatment for obesity and its comorbidities. The underlying mechanisms of weight loss after obesity surgery are not yet fully understood. Changes to taste function could be a contributing factor. However, the pattern of change in different taste domains and among obesity surgery operations is not consistent in the literature. A systematic search was performed to identify all articles investigating gustation in human studies following bariatric procedures. A total of 3323 articles were identified after database searches, searching references and deduplication, and 17 articles were included. These articles provided evidence of changes in the sensory and reward domains of taste following obesity procedures. No study investigated the effect of obesity surgery on the physiological domain of taste. Taste detection sensitivity for sweetness increases shortly after Roux-en-Y gastric bypass. Additionally, patients have a reduced appetitive reward value to sweet stimuli. For the subgroup of patients who experience changes in their food preferences after Roux-en-Y gastric bypass or vertical sleeve gastrectomy, changes in taste function may be underlying mechanisms for changing food preferences which may lead to weight loss and its maintenance. However, data are heterogeneous; the potential effect dilutes over time and varies significantly between different procedures.

## 1. Introduction

In modern life, food intake no longer fulfils the mere purpose of nourishment and sustaining function and physiological integrity, but also serves to satisfy hedonic desires. Hedonic food intake, i.e., the consumption of food for pleasure and palatability fuelled by flavour, is the result of a complex interplay of sensory perception in which taste comprises an important component [1,2].

### 1.1. Physiology of Taste

Sensory inputs are detected when they activate taste receptor cells (TRCs) in the taste buds located in the epithelium of the tongue, epiglottis and palate [3]. TRCs respond to chemical stimuli dissolved in saliva, which allows the detection of the five distinct taste modalities: salty, sweet, bitter, sour and savoury [4,5,6]. The primary receptors for sweet, savoury, and bitter stimuli are G-protein coupled receptors expressed in membranes of taste receptor cells, whereas the primary receptors for salty and sour taste are currently believed to be ion channels [3].

In addition, recent evidence suggests that dietary fat, especially free fatty acids, may be perceived chemically in taste bud cells as well as the five basic tastes. However, long chain fatty acids alone are flavourless, and it is not clear whether the chemical reception of fats resembles the other five taste stimuli, as their perception depends on smell and mouth-feel [7].

TRC activation leads to peptide and neurotransmitter release into afferent fibre terminals of cranial nerves VII, IX and X (facial, glossopharyngeal and vagus, respectively). These in turn convey information to the central nervous system, through the nucleus tractus solitarius in the brainstem to the thalamus and insula [4,8]. The primary gustatory cortex is the anterior insula and the frontal operculum. The insula also receives inputs from other sensory modalities, including pain, temperature, touch and olfaction [4,8].

These pathways help identify food taste qualities and modulate eating behaviour. These processes are categorized into three major domains [1,9]:

1. The sensory domain is concerned with the detection, recognition and perception of the intensity of a stimulus, e.g., does this cake taste sweet and how sweet? The detection threshold is defined as the minimum concentration at which a participant identifies a taste stimulus different from water, whereas the recognition threshold is defined as the minimum concentration at which a participant recognizes the quality of the taste stimulus. Taste intensity can be defined as the magnitude of a quality of the taste; it affects the liking of foods, and determines food choice and consumption [10].
2. The hedonic domain refers to the reward driving ingestion of a stimulus, e.g., how much do I want this cake and how much do I like it when I eat it?
  Food reward can be divided into appetitive and consummatory components. Appetitive reward reflects the effort made to pursue the desired food, and consummatory reward is the pleasure derived upon ingesting the food [11]. The two reward behaviour components can be studied in isolation and combination, depending on the required outcome. The direct behavioural method used to study appetitive food reward is the progressive ratio task (PRT) [12,13]. In the PRT, the subject must work for a rewarding stimulus; for example, this could involve clicking a computer mouse several times which measures motivational incentive or reward strength of a stimulus [12]. Consummatory behaviour can be assessed using a taste reactivity test [14]. This test has not been used in human studies but only in animal studies. The stimulus is delivered directly to the oral cavity, and the facial reactions are videotaped [14]. The common positive responses in the animals included paw licking and tongue protrusions, while the common negative response included chin rubbing and gape [15]. The brief access test is another method that can measure both components of the reward domain. This test has also been used in animal models only. A small amount of the taste stimulus is presented for a short duration (around 10 s), and a lick monitoring system (gustometer) is used to measure the animal licking responses [16]. It measures the amount of effort the animal makes to approach the stimulus, i.e., appetitive reward. It also reflects the consummatory reward domain by measuring the repetitive licks per unit over the test duration [16].
3. The physiological domain comprises the body’s reactions to sensing, i.e., the smell or sight of food. These reactions lead to the activation of pathways to help with digestion and maintenance of homeostasis. Salivation is the most apparent response and can be triggered by the mere thought of food but usually increases when food is present in the oral cavity [17]. There are links between the physiological and reward domains. It has been reported that people with obesity have a higher rate of salivation to food cues [18,19]. People with obesity also have a slower habituation rate (reduced salivation to the same food within a meal), possibly causing more food intake, as saliva helps dissolve food molecules and improves the ability to taste [18,20,21,22]. Another taste-related cephalic response is pre-absorptive insulin release, studied extensively in animals [23,24,25,26] and humans [27,28,29,30], however, not in relation to taste.

### 1.2. Taste and Obesity

Taste is one of the factors that are responsible for eating rate, as it is related to the duration of oral exposure to food, and thereby affects satiation [31]. The sense of taste plays an essential role in eating behaviour, as it contributes to food choice, energy intake, and, hence, body weight regulation [32,33]. Taste buds play a crucial role in how food-related signals are transmitted to the brain, particularly in priming the body for digestion during the cephalic phase, i.e., the start of the digestive process [34]. The development of obesity is associated with a significant reduction in taste buds [35,36,37] and impaired taste bud sensitivity [38,39,40]. In addition, genetic variation in taste receptors has been linked to body weight regulation [37]. Therefore, taste mechanisms could become targets for the development of treatments for obesity. [41,42]. A substantial amount of work has been performed on the impact of obesity surgery on taste function.

Vertical sleeve gastrectomy (VSG) and Roux-en-Y gastric bypass (RYGB) surgery are the two most commonly performed procedures, and the most effective treatments, for obesity [1,43]. We are only beginning to understand some of the complex mechanisms by which RYGB and VSG reduce hunger and increase satiety, changes in food preferences, and psychological aspects of eating behaviour [44]. Moreover, patients’ taste and food choices appear to change after surgery [45,46]; however, there is substantial heterogeneity in the results of the available studies on taste function.

There are three systematic reviews on the gustatory changes following obesity surgery [3,8,47]. However, they lacked clear inclusion and exclusion criteria, and were restrictive in scope, i.e., offering only narrative description, or only reporting studies assessing sweet taste or taste sensitivity.

This systematic review aims to pool the data available from the literature on the impact of obesity surgery on the sensory, reward and physiological domains of taste function. Any changes in taste after obesity surgery that are identified will be discussed in the context of changes in food choices and eating behaviour.

### 1.3. Aims and Objectives

This systematic review aims to assess studies examining the research question: does obesity surgery alter taste function in humans with obesity?

The primary aim is to review:

- Collated results from individual studies in the literature reporting taste in cross-sectional and longitudinal studies of obesity surgery.

Secondary aims are to review:

- How heterogeneity in study methodology, design, protocol and analysis might explain differences between studies;
- Differences between results particularly in terms of type of obesity surgery.

## 2. Materials and Methods

A systematic review of studies examining the impact of obesity surgery on taste function using direct sampling of taste stimuli or food.

### 2.1. Inclusion and Exclusion Criteria

The studies selected for the review included the following criteria.

#### 2.1.1. Inclusion Criteria

- Studies published in English.
- Articles published between January 1980 to May 2021.
- Studies conducted on adolescents or adults aged ≥15 years of either gender.
- Participants in the intervention group diagnosed with obesity with BMI >30 kg/m^2^.
- Assessments of obesity surgery (RYGB or single anastomosis gastric bypass, VSG, adjustable gastric banding (AGB), vertical banded gastroplasty, pancreato-biliary diversion).
- Studies using physical taste stimuli or food as a measurement scale to assess taste change in the sensory domain (detection and/or recognition), reward domain (consummatory and/or appetitive), or physiological domain (differences in salivation).
- Studies that investigated the effect of obesity surgery on taste function either by comparing pre- vs. post-obesity surgery or cross-sectional including post-obesity surgery in patients and un-operated control groups.

#### 2.1.2. Exclusion Criteria

- Studies conducted on animals.
- Reviews, editorials, letters and meeting abstracts.
- Articles assessing non-obesity procedures (e.g., gastrectomy secondary to gastric cancer); patients with obesity who did not undergo surgery or patients pre-surgically without post-surgical follow-up; and articles assessing satiety or hunger instead of gustation.

### 2.2. Database Search

An electronic database search was conducted to select articles to form the evidence base for this review. A comprehensive search across multiple databases and journals was performed using PubMed and Web of Science, and PsycINFO, MEDLINE and EMBASE databases within OVID. Reference lists from individual papers and relevant review articles were scrutinised further to include eligible studies. Two independent reviewers screened the titles and abstracts of the identified records for selection. A third investigator resolved any conflict in the study selection. There were 6 out of 132 (5.3%) articles that the third reviewer was asked to assess for eligibility. Additionally, any article relevant to the topic of interest but not found in the search or reference tables was included. Articles published up to 1 May 2021 were considered for inclusion.

### 2.3. Keywords/Terms Used

Population-based (‘bariatric surgery’ OR ‘obesity surgery’ OR ‘metabolic surgery’ OR ‘gastric bypass’ OR ‘Roux-en-Y’ OR ‘RYGB’ OR ‘single anastomosis gastric bypass’ OR ‘sleeve gastrectomy’ OR ‘AGB’ OR ‘gastric band’ OR ‘vertical band gastroplasty’ OR ‘duodenal switch’ OR ‘pancreato-biliary diversion’) in combination with taste change (‘taste’ OR ‘gustation’ OR ‘reward’ OR ‘consummatory’ OR ‘wanting’ OR ‘liking’ OR ‘appetitive’ OR ‘food preference’ OR ‘salivation’ OR ‘cephalic phase’) including their cognates and synonyms.

### 2.4. Data Extraction

The core data generated from every article were: author’s name, publication year, journal, country, demographic characteristics of participants (such as patients’ age, gender, ethnicity, and baseline/post-surgery body mass index), study design, sample size, type of obesity surgery, post-operative weight loss, taste measurement tool, type of post-operative taste change (i.e., the taste modality and domain), time elapsed since surgery, compliance rate and limitations of the study.

### 2.5. Outcome Measure

The primary outcome for our systematic review comprised changes in taste function post-obesity surgery, in terms of detection, recognition, intensity, and reward value of food based on the five major taste modalities (sweet, salty, bitter, savoury and sour), and physiological changes (habitual salivation).

## 3. Results

A total of 17 studies encompassing 613 patients who underwent bariatric surgery for obesity were included in the systematic review. Of these patients, 336 underwent RYGB, 243 VSG and 34 AGB surgery.

### 3.1. Study Design

Of the 17 included studies, 16 (94.1%) were of a longitudinal design and one (5.9%) was cross-sectional. Among the different types of surgical intervention, 11 assessed the effect of RYGB, six assessed the effect of VSG, and one assessed the effect of AGB. One assessed VSG and RYGB as one combined group, while one study assessed VSG, RYGB and AGB as one combined group.

#### Search Results and Selection of Studies

**Figure 1:**
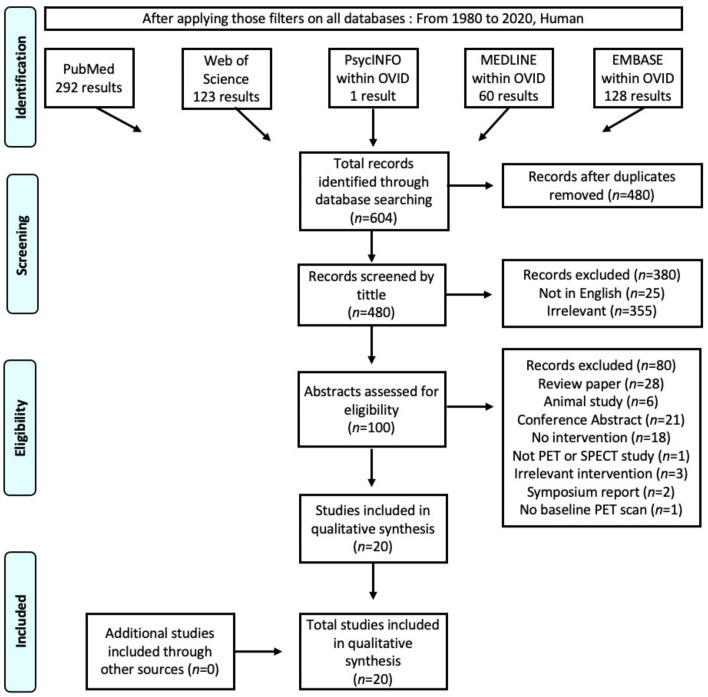
Flowchart illustrating the number of records identified and filters used in the review.

The selection of studies is illustrated in Figure 1. Using the keywords described above, 3323 articles were identified and 563 of these remained after duplicates were removed. Of these, 431 were excluded, with only 17 studies meeting the inclusion criteria (Figure 1).

### 3.2. Demographic Data

Patients’ mean age ranged from 15 to 56 years [13,48]. Preoperative mean BMI varied between 41.3 to 60.8 kg/m^2^ [11,49] and the post-operative weight loss reported was between 11.9% and 37.8% of the total body weight [50,51]. The number of participants per study ranged from 16 [52] to 136 [53]. Four studies included only female participants. Ten studies had 50% or greater, and one had fewer than 50% female participants. One study did not report the sex of participants.

Five out of 17 studies (29.4%) reported participants’ ethnic groups, and out of the 188 participants in these studies, 131 (69.7%) were White. Seven studies were conducted exclusively on patients without type 2 diabetes mellitus (T2DM); five studies included participants both with and without T2DM, and five studies did not report T2DM status.

Twelve studies included groups of fewer than 10 participants, eight included groups between 10 and 20 participants, and nine included groups of 20 participants or more. The total number of studies here is more than the number of papers as some studies had more than one intervention group.

### 3.3. Study Protocols

#### 3.3.1. Nutritional Status before the Taste Test

In four out of 17 (23.6%) studies, participants were assessed after a 1 h fast [51,53,54,55], three (17.6%) studies after fasting 2 to 3 h [13,49,56] two studies (11.8%) after fasting 4 to 5 h [11,57] and five (29.4%) studies after overnight fasting [46,48,50,58,59]. Three studies (17.6%) did not report nutritional status [45,52,60].

#### 3.3.2. Time since the Intervention

In the longitudinal studies, taste was measured as early as 1 month [11,52,54] to 18 months post-surgery [51]. The cross-sectional study carried out the experiment 16.8 ± 14.5 months after surgery [49].

### 3.4. Findings

#### 3.4.1. Sensory Domain

There are several methods used to assess detection and recognition thresholds and intensity perception. Table 1 shows the most commonly used, and explains the procedure for each method.

##### Taste Detection after Obesity Surgery

Demographic and methodological data for individual detection studies are given in Table 2.

Nine studies measured the effect of obesity surgery on taste detection. Eight of them examined the effect of obesity surgery on sweet taste, three on sour taste, six on salty taste, two on savoury taste and three on bitter taste. The total number of studies here is more than the number of papers, as some studies investigated the effect of obesity surgery on more than one taste modality.

Regarding sweet taste, studies that investigated the effect of obesity surgery early post-intervention (1 to 2 months) observed an improvement in taste detection [50,52]. Taste detection was improved 1 month after RYGB using the 3-AFC (*n =* 6) and 2-AFC (*n* = 21) [51,52]. Studies that investigated the effect of obesity surgery after this initial period observed different results. No change was observed 3 months after RYGB using the 3-AFC (*n* = 6), or 6 months after RYGB using the 2-AFC (*n* = 21) [51,52], nor was a change observed 3 and 12 months after VSG using the method of constant stimuli (*n* = 14) [48]. Similarly, no change was seen after 20% weight loss (around 6 months) following RYGB or AGB (combined as one group; *n* = 17 RYGB, *n* = 10 AGB), nor after RYGB or VSG (*n* = 23 RYGB, *n* = 8 VSG) using the 2-AFC [46,58]. However, studies that used the Burghart taste strip method observed an improvement in taste detection 1 and 3 months after VSG (*n* = 52), and 6 months after RYGB, VSG and AGB (combined as one group) (*n* = 6) [45,54].

Three studies observed an improvement in sour taste detection: one of them at 1, 2 and 3 months after RYGB using the 3-AFC (*n* = 6); another at 1 and 3 months after VSG using the Burghart taste strip method (*n* = 52); and the third at 6 months after RYGB, VSG and AGB (as one group) (*n* = 44) using the Burghart taste strip method [45,52,54].

A trend of improvement in salty taste detection was observed 1, 2 and 3 months after RYGB using the 3-AFC (*n* = 6), and 3 months after RYGB and single anastomosis gastric bypass (combined as one group) using the 3-AFC method (*n* = 19) [52,60]. No change in salty taste detection was observed at 20% weight loss after RYGB and AGB surgery (combined as one group; *n* = 17 RYGB, *n* = 10 AGB), or post RYGB or VSG (*n* = 23 RYGB, *n* = 8 VSG) using the 2-AFC method [46,58]. As for sweet taste, studies that used the Burghart taste strip observed a significant improvement in salty taste detection 1 and 3 months after VSG (*n* = 52), and 6 months after RYGB, VSG and AGB (combined as one group; *n* = 3 RYGB, *n* = 37 VSG, *n* = 4 AGB) [45,54].

Two studies observed no change in savoury taste detection at 20% weight loss after RYGB and AGB surgery (combined as one group; *n* = 17 RYGB, *n* = 10 AGB) or after RYGB or VSG (*n* = 23 RYGB, *n* = 8 VSG) using the 2-AFC method [46,58].

A significant improvement in bitter taste detection was observed in all three studies that measured it. One study used the 3-AFC method at 1, 2 and 3 months after RYGB (*n* = 6) [52]. Two studies used the Burghart taste strip method at 1 and 3 months after VSG (*n* = 52), and after RYGB, VSG and AGB (combined as one group; *n* = 3 RYGB, *n* = 37 VSG, *n* = 4 AGB) [45,52,54].

##### Taste Recognition after Obesity Surgery

Demographic and methodological data for individual studies are given in Table 3.

Five longitudinal studies examined the effect of obesity surgery on taste recognition thresholds, and one cross-sectional study compared RYGB with VSG surgery [11,45,49,51,52,54]. All of the studies investigated the effect of obesity surgery on sweet taste, four studies on sour, four on salty and five on bitter taste. The total number of studies here is more than the number of papers, as some studies investigated the effect of obesity surgery on more than one taste.

Regarding sweet taste, one study observed a significant improvement in taste recognition 1.5 and 3 months after RYGB surgery using the 2-AFC method (*n* = 14) [11]. Studies that used the Burghart taste strip method observed a significant improvement in sweet taste recognition 1 and 3 months after VSG (*n* = 52), and 6 months after RYGB, VSG and AGB (combined as one group; *n* = 3 RYGB, *n* = 37 VSG, *n* = 4 AGB) [45,54]. Another study only observed a trend of improvement 1 and 2 months after RYGB surgery, with no change in taste recognition 3 months after RYGB surgery compared with pre-surgery using the 3-AFC (*n* = 6) [52]. By contrast, no change was observed 1.5 months after RYGB surgery, while an improvement in taste recognition was observed 6 months after RYGB using the 2-AFC (*n* = 21) [51]. In the cross-sectional study, no difference was observed in sweet taste recognition 16 months after RYGB (*n* = 9) when compared with 22 months after VSG (*n* = 12) [49].

Regarding sour taste, studies that used the Burghart taste strip method observed a significant improvement in sour taste recognition 1 and 3 months after VSG (*n* = 52), and 6 months after RYGB, VSG and AGB (combined as one group; *n* = 3 RYGB, *n* = 37 VSG, *n* = 4 AGB) [45,54]. One study observed variable results over time in sour taste. The recognition threshold increased (i.e., worsening in taste recognition) non-significantly at 1 month after RYGB; however, the direction was changed and they observed a significant reduction in recognition threshold (i.e., improvement in taste recognition) 2 months after RYGB and a trend of improvement 3 months after surgery compared with before RYGB surgery using the 3-AFC (*n* = 6) [52]. Sour taste recognition thresholds were worse in patients 16 months after RYGB (*n* = 9) compared to patients 22 months after VSG (*n* = 12) using the 3-AFC [49].

Salty taste recognition tended to improve 1, 2 and 3 months after RYGB using the 3-AFC method (*n* = 6) [52]. Significant improvement in salty taste recognition was also observed using the Burghart taste strip method 1 and 3 months after VSG (*n* = 52), and 6 months after RYGB, VSG and AGB (as combined group) [45,54]. No difference in salty taste recognition was seen in patients after RYGB (*n* = 9) compared to patients after VSG (*n* = 12) using the 3-AFC test [49].

A significant improvement in bitter taste recognition was seen 1 and 3 months after VSG (*n* = 52), and 6 months after RYGB, VSG and AGB (combined as one group; *n* = 3 RYGB, *n* = 37 VSG, *n* = 4 AGB) using the Burghart taste strip method [45,54]. A similar finding was observed 1, 2 and 3 months after RYGB using the 3-AFC method (*n* = 6) [52], while another using the 2-AFC method observed no change 1.5 and 3 months after RYGB (*n* = 14) [11]. No difference in bitter taste recognition was seen in patients 16 months after RYGB (*n* = 9) compared to patients 22 months after VSG (*n* = 12) [49].

##### Taste Intensity after Obesity Surgery

Demographic and methodological data for individual studies are given in Table 4.

All studies used a series of concentrations for each stimulus and subjects rated the perceived intensity of the stimulus using the generalised labelled magnitude scale. No change in intensity perception was observed after 20% weight loss either after RYGB (*n* = 23) or after VSG (*n* = 8) in sweet, salty and savoury taste tests using a series of concentrations of sucrose, glucose, sodium chloride and monosodium glutamate presented in ascending concentrations [58].

Similarly, no differences were observed in perceived sweetness of glucose and sucrose, savoury taste of monosodium glutamate and saltiness of sodium chloride presented in random order at 20% weight loss following RYGB and AGB surgeries (as combined group), although sucrose was perceived as 7% less sweet [46]. One study compared participants 1 year after RYGB (*n* = 52) or VSG (*n* = 34) surgery (as combined group) with an un-operated obese control group (*n* = 50), and observed no differences in taste perception of sweet, sour, salty and bitter taste between the two groups using taste strips presented in ascending concentration (sucrose, citric acid, sodium chloride and quinine hydrochloride) [53].

#### 3.4.2. Reward Domain

There are several methods used to assess the reward value of a taste. Table 5 lists the most common methods and the procedures used to assess the reward value of a given taste.

##### Appetitive Reward

Two longitudinal studies used the PRT to study the effect of obesity surgery on the appetitive reward domain [13,56]. In one study, a decrease in reward value (breakpoint) for a sweet and fat stimulus, but not for vegetables, was observed 2 months after RYGB [13]. In the other study, a decrease in reward value for sweet/fat stimulus was seen 3 and 12 months after VSG in an adolescent cohort [56]. The breakpoint for a sweet/fat stimulus after RYGB surgery increased with acute suppression of the elevated post-prandial satiety gut hormones PYY and GLP-1 using the somatostatin analogue Octreotide, implicating these hormones in the post-operative reduction of appetitive reward [55].

##### Consummatory Reward

Nine studies investigated the effect of obesity surgery on the consummatory reward domain [46,49,50,51,53,57,58,59,60]. Eight examined sweet taste, one sour and bitter taste, two salty taste, and three savoury taste. Different methods were used to assess consummatory reward, and various scales were used to assess the reward value of taste.

One study observed a reduction in the consummatory reward value of sweet after RYGB (*n* = 17), but not AGB (*n* = 10), as measured by rating pleasantness on a generalised labelled hedonic scale (gLHS). The study also employed a 2-AFC which revealed a preference for lower sucrose concentrations after surgery, with no differences between RYGB and AGB procedures [46]. In another cohort of patients after 20% weight loss following RYGB (*n* = 23) and VSG (*n* = 8), using the same methodology, there was a reduction in consummatory reward for sweet taste, but no change in sucrose preference [58].

A cross-sectional study found lower hedonic ratings across a range of concentrations of sweet solutions for patients after RYGB (*n* = 9) than after VSG (*n* = 12), using a 9-point hedonic category scale to rate nine different concentrations of sweet solutions [49]. Similarly, patients after RYGB (*n* = 21) rated the taste of ice cream (sweet/fat stimulus) as less pleasant on a visual analogue scale (VAS) than after AGB (*n* = 19), despite similar ratings of intensity [59]. Interestingly, this was accompanied by and correlated with lower reward system activation during evaluation of high-energy food pictures, using functional magnetic resonance imaging (fMRI), after RYGB than after AGB surgery, indicating a positive relationship between consummatory and anticipatory (cue reactivity) reward responses to high-energy foods [59].

By contrast, no change in the consummatory reward value of sweet stimuli was found at 1.5, 6 and 18 months after RYGB (*n* = 21) and VSG (*n* = 8) surgery (as a combined group), as assessed using 9-point hedonic category scale ratings for different concentrations of sucrose either in apple juice or tomato soup [51]. Similarly, a combined group of patients after RYGB (*n* = 52) and VSG (*n* = 34) showed no differences in “pleasure” rating of sucrose using taste strips with a gLHS compared with un-operated participants with obesity [53]. Only one study used VAS to test the concentration of sucrose that was “just about right” in patients before and 2 months after RYGB and normal-weight controls, with no differences in the “just about right” sucrose concentration between or within groups [50].

Interestingly, one study observed a positive correlation between consummatory ratings for sucrose-sweetened mixtures before surgery with greater weight loss 6 months after RYGB, but not VSG [57]. The participants rated their preferences for taste mixtures (milk with different sucrose and fat concentrations) using VAS.

Only one study compared consummatory reward for sour and bitter tastes in participants 12 months after RYGB (*n* = 52) and VSG (*n* = 34) surgery (as a combined group) with an unoperated obese control group (*n* = 50), and observed no differences in pleasure rating for sour or bitter taste [53].

A combined group of patients who underwent RYGB or single anastomosis gastric bypass (*n* = 19) rated their preferences for cream soups with varying amounts of salt prior to and 3 months after surgery [60]. No change in the preference ratings on a 9-point hedonic category scale were seen. Similarly, no difference was observed in the pleasantness of salty taste strips between patients 1 year after RYGB (*n* = 52) or VSG (*n* = 34) surgery (as a combined group) and unoperated patients with obesity (*n* = 50) using the gLHS [53].

Preference for savoury taste after RYGB and AGB was investigated in a study using a 2-AFC tracking procedure for monosodium glutamate solutions. No changes in preference were observed at 20% weight loss after RYGB (*n* = 17) and AGB (*n* = 10) surgery (as a combined group) compared to before surgery [46]. Similarly, this group found no change in savoury preference using 2-AFC after 20% weight loss following RYGB/VSG surgery [58]. Additionally, no change in liking was seen at 1.5, 6 and 18 months after RYGB (*n* = 21) and VSG (*n* = 8) surgery (as a combined group) using the 9-point hedonic category scale for different concentrations of monosodium glutamate in tomato soup [51].

#### 3.4.3. Physiological Domain

No study has investigated the effect of obesity surgery on the physiological component of taste, i.e., the cephalic phase responses. However, several studies observed an improvement in salivation flow rate after obesity surgery, but they did not use taste/food stimuli, and thus were not included in this systematic review [64,65,66,67].

## 4. Discussion

### 4.1. Sensory Domain

In the sensory component, all studies investigating the taste detection threshold after obesity surgery observed an improvement in sweet taste detection 1 and 2 months post obesity surgery [50,51,52,54]. However, only studies that used Burghart taste strip tests observed an improvement in sweet taste detection after 3 to 6 months [45,46,48,51,54,58]. A similar pattern was observed for salty taste. Only one study investigating the effect of obesity surgery on sour and bitter taste found an improvement in taste detection in those taste modalities. By contrast, all of the studies that investigated savoury taste observed no change in savoury taste detection. Moreover, studies that examined taste recognition thresholds found conflicting results for sweet, salty, sour and bitter taste, while all of the studies, except for one, found no effect of obesity surgery on taste intensity perception.

Both studies of appetitive behaviour found a decrease after RYGB and VSG [13,56]. However, the findings on the consummatory reward value of different taste modalities after surgery were not consistent.

The short-term improvement in sweet and salty taste detection in the first 2 months following surgery may be due to decreased sensory experience of the sweet and salty taste caused by reduced food intake. That is to say, tasting these types of foods less frequently following surgery may cause increased sensitivity (i.e., decreased detection and recognition thresholds) to the tastes. Another explanation may be that repeated testing increases familiarity with and improved ability to identify the taste stimuli during the assessment. Diabetes mellitus or depression are known to cause gustatory dysfunction for sweet taste [68]. Inclusion of patients with T2DM and depression may, therefore, be a confounder that contributes to the heterogeneous findings. However, only studies that used the Burghart taste strip method demonstrated improvement in sweet taste detection and recognition beyond 3 months after surgery. This may be due to some of the limitations of using taste test strips. For example, the tastant on the strip needs to dissolve in saliva; therefore, any changes to saliva production after surgery or weight loss could affect the results of the taste strip scores [45,54,69].

An improvement in sweet taste detection was observed following 3 months on a low-calorie diet [70]. No study in this review had a dietary weight loss intervention control group, and so it cannot be determined whether any observed taste changes were due to weight loss or the surgeries themselves.

Regarding savoury taste, both studies that observed no improvement in savoury taste detection investigated the effect of obesity surgery after 20% weight loss. As observed with other taste modalities, it is possible there are improvements in savoury taste detection shortly after surgery; however, no study in this review investigated that time frame for savoury taste.

Several limitations in the included studies make it difficult to reach definitive conclusions. First, some studies used very small tastant drops that cause limited receptor stimulation, which may limit the sensation of the taste. Second, most of the included studies lacked a control group for the effects of repeated testing. Third, the concentrations of the tastants used varied among different studies. In addition, variation in the nutritional state and recent dietary phase (e.g., early post-operative liquid diet) of participants at the time of testing, the methodologies employed to measure taste thresholds, methods of presenting the stimuli, sex differences and time of testing after surgery may all have contributed to the observed heterogeneity.

### 4.2. Reward Domain

#### 4.2.1. Appetitive Reward

There are only two studies, one conducted in adults [13] and one in adolescents [56], that have used direct measurement (PRT) to assess changes in the appetitive domain after obesity surgery. Both studies observed a decrease in the reward value of a sweet/fat tastant [13,56], but not vegetables [13].

The findings from these direct measurements are supported by studies using indirect food scale questionnaires. These studies found decreases in wanting scores for sweet foods such as chocolate, cakes, biscuits, cookies, fruit juice and soft drinks in patients after RYGB [71], after RYGB vs. unoperated controls with obesity [72], and after AGB vs. unoperated controls with obesity [73]. However, the food scale questionnaires do not give the actual consumptive behaviour and do not specifically measure the change in taste.

Using animal models of obesity surgery expands our understanding of the mechanisms that cause changes in eating behaviour. In animal studies, the effect of RYGB and VSG on the appetitive responsiveness to sweet taste was studied using brief access tests and PRTs in rats [74,75,76,77]. However, results were mixed, with some studies reporting no change while others showed an increase in appetitive responsiveness to sweet taste. Two studies, one after RYGB and another after VSG, did not show lower breakpoints in the PRT for sucrose in rats than in sham-operated rats [74,77]. Interestingly, another study using a brief access test observed that rats after RYGB demonstrated a doubling of the breakpoint for sweet stimuli [76]. Another study observed a decrease in licks for the highest three concentrations of sucrose (0.25–1.0 mol/L) in rats after RYGB compared to sham-operated rats [75]. However, considerably higher mean licks for the sucrose concentrations were recorded compared with water or with the low sucrose concentrations, suggesting that the rats still worked harder for the sweeter stimuli. The benefit of the brief access test is that it minimises any post-ingestive effects of the taste stimuli, as only small amounts are ingested. However, the inconsistencies between human and animal studies are interesting and may be due to several reasons. Significant differences between humans and rats in their oral mucosae may affect taste function [78], in addition to differences in gastrointestinal tract absorptive capacity that may alter post-absorption phenomena [79].

Additionally, participants may behave in a specific way to “please” the researcher, which may explain why human findings are positive, while animal models are inconclusive. Moreover, rats are usually studied during the maintenance phase of weight loss, while human studies are generally during the negative energy balance phase post-surgery, and this may differentially affect appetitive reward.

#### 4.2.2. Consummatory Reward

Among the five main taste modalities (sweet, bitter, salty, savoury and sour), changes in sweet taste after obesity surgery were the most commonly measured. The results for sweet taste exhibit some inconsistency. Some studies observed a reduction in consummatory reward of sweet taste [46,57,58,59], while others did not [49,51,53,80]. The methods used in these studies varied considerably, as did the concentrations of sweet solutions.

Another possible factor is the failure to identify ‘sweet-liker’ phenotypes amongst participants that could influence the outcomes. There are inter-individual variations in hedonic responses to sweet taste. Humans exhibit different response patterns: a ‘sweet-liker’ phenotype characterized by a rise in liking as concentration increases and a ‘sweet-disliker’ phenotype characterized by a decline in liking as concentration increases [81]. The substance most commonly used to investigate the affective reactions elicited by sweetness is sucrose. A study population consisting predominantly of ‘sweet-dislikers’ or ‘sweet-likers’ could skew results. A sweet-liking phenotype has been associated with different hedonic responses to sweet, and potentially this may relate to how such patients benefit from obesity surgery [57]. A positive correlation between “liking” sweet taste pre-surgery with weight loss after surgery has been reported. These results are consistent with recent studies that tested liking ratings for sucrose-sweetened mixtures containing fat. They show that in patients undergoing RYGB but not VSG surgery, a higher preoperative preference for sucrose-sweetened combinations predicted post-operative weight loss [57]. Moreover, it was observed that even a slight reduction in sweet taste palatability score after surgery correlated positively with weight loss [53,57].

Despite inconsistencies, the literature regarding RYGB and VSG surgery has broadly described a reduced food preference for sugary food, without pinpointing taste as a mediator for this change. It is reassuring that the change in consummatory reward is in line with reports of reduced preference for sugary food. Patients who have undergone RYGB or VSG prefer to eat food low in sugar compared with pre-surgery [82,83,84,85,86]. Patients post-RYGB or -VSG also prefer to eat foods lower in sugar than patients post-AGB or vertical banded gastroplasty [73,87,88]. However, all of those studies used indirect measurements such as questionnaires and food recall.

The two-bottle preference test found a significant decrease in preference for sucrose relative to water 4 weeks after RYGB in rats [80]. The lick responses of rats to sucrose decreased across a range of concentrations (0.01, 0.03, 0.10, 0.3, 1.0, 1.5 M) 3 weeks post-RYGB compared to pre-surgery in a brief access test. [89]. The number of licks decreased only for the higher end of a range of sucrose concentrations (0.25–1.0 M) in RYGB compared to sham-operated rats [75]. There was no effect from RYGB on water licks, indicating that the effect was specific for the sweet stimulus and not a general overall decrease in licking behaviour. By contrast, no change was observed in licking of sucrose using a similar brief access test design in another study [76]. The lack of consistency between the rodent studies may be due to the differences in the geometry of the gut remodelling, time since surgery, concentrations of the stimuli and different nutritional status when the tests are conducted, i.e., subjects are fed or fasted.

The improvement in taste function after obesity surgery was observed for sweet taste. Post-ingestive effects of high sugar/fat nutrients resulting in conditioned taste avoidance may partially explain this observation. This was consistent with one study, which reported no difference in appetitive or consummatory behaviour in the brief access test (measures liking without the effect of post-ingestion) for intralipid between rats after RYGB versus sham surgery [88]. In a separate study, RYGB rats did decrease their preference for intralipid relative to water using the two-bottle preference test [50]. This may lead to the hypothesis that changes in food preferences after obesity surgery may not result from changes in the hedonic value of food but rather from learning that too much sugar/fat may have negative visceral consequences. The biological system is geared towards learning when consumption causes post-ingestive malaise. After obesity surgery, food containing sugar/fat may be perceived as “harmful due to negative post-ingestive sequelae” [16].

The dumping syndrome, which is a group of symptoms, such as diarrhoea, nausea, and feeling tired after a meal, that are caused by rapid gastric emptying after RYGB surgery, has been proposed to induce these changes in food preference [90]. Post-ingestive symptoms have been reported by 15–70% of patients after RYGB surgery, which is believed, but not proven, to result in altered food preferences [91,92,93]. When dumping syndrome was first recognised, it was considered as a useful characteristic of RYGB surgery to ‘teach’ patients to evade energy-dense foods and thus consume fewer calories. Some studies reported that ‘sweet-eaters’ lost more weight after RYGB, as sweet and fatty foods induce the symptoms of the dumping syndrome and these patients would consume less of these types of foods [94,95]. Reduction in the consumption of sweet foods due to dumping syndrome does not appear to be due to classical conditioned food aversion, i.e., dislike of sweet foods, as most patients with severe dumping syndrome seem to still like the taste of sweet foods. Rather, conditioned food avoidance (i.e., liking but not eating) is a more plausible explanation. A distinction between these terms is important because avoidance implies that the palatability of sweet or fat still exists with small quantities consumed, but that the individual learns to stop eating earlier, as large quantities may have negative visceral consequences [96]. Fewer patients with VSG have been suspected of early dumping syndrome than after RYGB [97,98], which could explain why the reduction in consummatory reward was more pronounced after RYGB compared to VSG surgery.

### 4.3. Physiological Domain

No study has measured the effect of obesity surgery on cephalic insulin release. Measuring preabsorptive insulin release can be challenging and requires a large sample size and careful protocol consideration [30].

## 5. Limitation of the Included Studies

Several limitations make it difficult to compare the studies and draw a definitive conclusion from the available literature. Most studies lacked a non-surgical intervention, making it challenging to know if the effects result from weight loss per se or purely from the obesity surgery. In addition, most studies used liquid forms of stimuli lacking texture and smell to test the influence on taste perception and reward function. These liquids are not typically consumed in, and therefore, not representative of, everyday life.

Methodological heterogeneity across studies is evident and may result in inconsistent and sometimes contradictory results. Different surgeries may have differing effects on gut anatomy and physiology and taste domains. Some of the studies in this review investigated combined groups composed of patients who underwent different surgeries, which compounded the difficulty of determining the effect of each surgery. Additionally, the time elapsed between surgery and post-surgical testing appears to be relevant at least for some taste domains, and this varied between studies. Nutritional status affects taste perception, and the duration of fasting prior to the tests varied between studies from 1 h to overnight [99]. The range and intervals of concentrations of tastants, and the tools used to assess them also varied between studies, creating challenging nuances in interpretation, i.e., comparing preference to liking.

The existence of confounding factors in some studies may also affect their interpretation. Some studies did not include smoking in the exclusion criterion, despite the fact that smokers exhibit lower taste sensitivity than non-smokers [61,62]. It is well known that age impacts taste perception, sensitivity and preference [100,101,102]. A range of age groups were included in these studies, from adolescents to over 60 years old. In addition, some studies included participants with T2DM, which may affect taste [103]. Most of the studies did not assess the participants’ psychological status. There is consistent evidence that obesity is associated with depression and anxiety [104,105,106], which can affect taste function [107,108,109].

Participants were predominantly female, although the ratio varied between studies. Taste function varies between the sexes, and taste, particularly sweet taste, changes through the menstrual cycle [110,111]. This was not considered in the studies presented here. Moreover, more than 75% of the participants were White, limiting the generalisation of the results to other ethnic groups.

No human studies have evaluated the link between taste and actual food intake, the effect of obesity surgery on the physiological domain of taste, or measured the direct connection between the change of satiety hormones post-obesity surgery and changes in taste recognition, detection or sensitivity, though gut hormones such as PYY and GLP-1 have been implicated in the changes in appetitive reward [55]. 

A potential role for satiety gut hormones in post-surgery changes in taste is suggested by some human and animal studies. Lower ice cream pleasantness in patients after RYGB than after AGB surgery was accompanied by higher satiety gut hormones PYY and GLP-1 after RYGB surgery [59]. An improvement in taste detection of sweet flavours using 3-AFC has been seen after injection of the GLP-1 analogue liraglutide [112]. A reduction in wanting, a desire to eat something sweet, salty, savoury or fatty, using VAS ratings has been seen after 16-week treatment with once-daily liraglutide compared with placebo [113]. Interestingly, preclinical research has implicated a direct role of GLP-1 in the gustatory coding of the tongue: (i) GLP-1 is locally synthesized in subpopulations of taste bud cells, (ii) the GLP-1 receptor is present on the gustatory nerve fibres in close proximity to GLP-1-containing taste bud cells, and (iii) this paracrine GLP-1 signalling is specifically involved in the perception of sweet taste [114,115,116,117]. Mice lacking the GLP-1 receptor have reduced taste sensitivity to both nutritive (sucrose) and non-nutritive sweeteners (sucralose) [114,115] and display hypersensitivity to the umami tastant MSG with a moderate increase in sensitivity to the sour tastant citric acid. This indicates that GLP-1 produced in taste cells plays an important role in modulating taste sensitivity for sweet and umami modalities [118]. Several animal studies have reported a dose-dependent aversion to sweet taste after administration of PYY [119,120].

Most studies on the role of taste in human nutrition are behavioural in nature, while neurobiological or physiological pathways remain largely unexplored. Few studies have examined the effects of obesity surgery on brain responses to sweet taste using fMRI, but detailed examinations of their neuroimaging findings were outside the scope of this review [121,122]. Interestingly, one study found a reduction in blood oxygen level dependent (BOLD) signal to chocolate milk taste (sweet, high fat) in the insula (which includes gustatory cortex) after RYGB surgery, but no taste ratings in any domains were measured [121]. Furthermore, this was attenuated by acute administration of the GLP-1 analogue exendin(9–39), indicating a potential role for the increased plasma GLP-1 after RYGB in these changes of sweet/fat taste responsivity.

Studies that fill the gaps in the literature will improve our understanding regarding the effect of surgery on taste function. For example, studies including a dietary intervention control group (e.g., very low-calorie diet) will enhance our understanding of the changes in taste resulting from weight loss compared to surgery. Studies that correlate the change in postprandial hormones with the difference in taste will improve our understanding of the changes (if any) resulting from hormonal change after surgery. Work is needed to interrogate the underlying mechanisms, phenotype patients who experience gustatory changes, and identify potential genetic and environmental factors that facilitate these changes. Moreover, studying cephalic phase responses in humans is another opportunity for future research to clarify further the nutrient signalling properties of taste and its relation to homeostatic and hedonic centres in the brain, as well as for further fMRI gustatory studies. Furthermore, most studies have been performed in laboratory situations. It is now time to translate these findings to real-life situations (e.g., living rooms and shopping malls) to investigate the effect of taste on our daily eating behaviour over more extended periods.

## 6. Conclusions

The studies presented in this systematic review found evidence supporting reported changes in taste sensory and reward domains, mainly sweet taste, and its relation to food preference following obesity surgery procedures. Changes included a short-term increase in detection and a decrease in preference for sweet taste. For the subgroup of patients who experience alteration in their food preferences after RYGB or VSG, changes in taste function may be contributory underlying mechanisms.

Although some patterns are emerging, there are several inconsistencies between human studies for varying reasons. First, factors such as BMI, age and sex can influence taste. Secondly, factors related to the surgery include differences in surgical techniques, weight loss, time from surgery, type of dietary advice and support provided. Thirdly, the differences in study design such as small sample size, combined surgical groups, statistical analysis, lack of adequate controls in longitudinal studies, and types of stimuli can all contribute to the inconsistencies.

More research is needed to address the limitations of previous studies. A greater understanding of the role of gustatory inputs in obesity and weight loss may provide an effective adjunct in finding effective medical treatment(s) for obesity.

## Figures and Tables

**Table 1:** Methods used to assess detection and recognition thresholds and intensity perception.

	Psychophysical Task	Stimulus Presentation	Stimulus
**Detection and recognition threshold**	**Constant stimuli**: In which taste stimuli are presented randomly and performance is assessed allowing for the derivation of a psychometric function. A “hit” is defined as when the participant correctly reported that the stimulus was different from water when stimulus was presented. A “false alarm” is defined as when the participant incorrectly reported that the stimulus was different from water when water was presented [50]. **2-Alternative Forced Choice (2-AFC):** Participants are asked to differentiate the tastant from water, in multiple trials. The test begins at a concentration at which the tastant can be detected/recognised. After two correct trials, the test moves to a lower concentration, while a single incorrect trial leads to a higher concentration. The threshold is calculated based on the reversal concentrations. This type of test is sometimes referred to as a staircase [61]. **3-Alternative Forced Choice (3-AFC):** Two samples of water and a sample of tastant are tasted at separate times for each trial. The detection threshold is the lowest concentration to receive two successive correct responses by the subjects just above the immediate lower concentration at which two successive incorrect responses are given or simply as the lowest concentration, the difference between the three drops can be detected. The recognition threshold refers to the concentration at which the subject can identify the taste quality [62]. **Burghart taste strip test:** The technique is based on strips made from filter paper which were impregnated with different taste solutions (four concentrations each for sweet, sour, salty and bitter). These strips are placed on the tongue and subjects are asked to identify the taste quality [63].	**Method of limits:** Target tastants are offered in increasing (ascending) or decreasing (descending) concentrations. The threshold refers to the minimum concentration for taste detection. **Method of constant stimuli:** Stimuli of variable concentrations are presented in a random order to participants. Concentrations range from those which are known to be subthreshold and supra-threshold. The threshold is the concentration of the stimulus, perceived in more than half of the presentations. **Adaptive method:** The initial stimulus is a known supra-threshold stimulus, and is followed by stimuli of concentrations that decrease in predefined steps. The series is stopped when the stimulus strength becomes subthreshold. Then, the step is halved and increasing concentrations are given until the subject perceives the taste again. This process is repeated several times, reversing each time, until the step size reaches the preset minimal value. With this method, the threshold value can be delineated very accurately.	Solutions Model foods/beverages Filter papers
**Intensity**	**Category scales:** These are numeric scales and generally comprise descriptors equally spaced across a line (e.g., ranging from “1 = no taste” to “9 = extreme taste”). Common examples are the 9-point scale or visual analogue scale (VAS). **General labelled magnitude scale (gLMS):** This comprises a top anchor (‘strongest imaginable sensation of any kind’), an opposite anchor (‘barely detectable’) and intermediate labels.	**Random order:** stimuli are presented in a random order of intensity. **Increasing concentrations:** stimuli are presented in an order of increasing intensity	Solutions Model foods/beverages Filter papers

**Table 2:** Key clinical parameters in the studies that investigated the effect of obesity surgery on detection threshold.

Author (year)	N	Group (s)	% Female	Age (y)	T2DM (%)	Time after Intervention (months)	Baseline BMI (kg/m^2^)	Weight Loss (% or kg)	Time Since Last Meal (h)	Taste Modality	Stimuli and Concentration	Methodology	Key Results (Post vs. Pre Surgery)
**Scruggs** **1994** [52]	6 10	RYGB NWC	100% 100%	34.1 ± 7.8 36.7 ± 6.9	?	1, 2, 3 n/a	44.2 ± 7.1 20.6 ± 1.9	15, 10, 7 kg n/a	?	sweet sour salty bitter	sucrose (6–5800 mM) HCl (0.5–500 mM) NaCl (6–6100 mM) urea (90–5000 mM)	3-AFC [62]	↑ SW ^a^, SO, SA, BI
**Bueter** **2011** [50]	99	RYGB NWC	88.9% 77.8%	?	?	2 2	44.8 ± 5.4 22.0 ± 3.0	14 kg	?	sweet	sucrose (2.1, 6.25, 12.5, 25, 50, 100 and 300 mM)	constant stimuli [50]	↑ SW
**Pepino** **2014** [46]	17 10	RYGB AGB	100% 100%	42.1 ± 8.4 46.8 ± 13.9	0 0	~20% WL ~20% WL	46.3 ± 7.7 48.5 ± 10.5	20.3 ± 3.0 kg 18.4 ± 2.0 kg	12 h	sweet salty savoury	sucrose, glucose, NaCl, and MSG: (1 to 1 × 10^−4^ M)	2-AFC [61]	↔ SW (s, g), SA, SAV ^b^
**Holinski** **2015** [45]	37/ 4/ 3 23	VSG/AGB/RYGB NOC	65.9% 56.2%	47.1 ± 9.8 39.5 ± 13.5	0 0	6 6	48.6 ± 7.5 23.4 ± 3.8	29.5 kg (20.6%) 0.1 kg (0.1%)	?	sweet sour salty bitter	sucrose (0.4, 0.2, 0.1 and 0.05 g/mL) citric acid (0.3, 0.165, 0.09 and 0.05 g/mL) NaCl (0.25, 0.1, 0.04 and 0.016 g/mL) quinine HCl (0.006, 0.0024, 0.0009 and 0.0004 g/mL)	Burghart taste strip [63]	↑ SW, SO, SA, BI ^b^
**Altun** **2016** [54]	52	VSG	57.7%	38.5 ± 9.4	?	1, 3	45.8 ± 7.2	1m: 25 ± 7.1% ^c^ 3m: 43.9 ± 10.3% ^c^	1 h	sweet sour salty bitter	sucrose (0.4, 0.2, 0.1, 0.05 g/mL) citric acid (0.3, 0.165, 0.09, 0.05 g/mL) NaCl (0.25, 0.1, 0.04, 0.016 g/mL) quinine HCl (0.006, 0.0024, 0.0009, 0.0004 g/mL)	Burghart taste strip [63]	↑ SW, SO, SA, BI
**Ekmekcioglu** **2016** [60]	19 29	RYGB/SAGB NOC	63.6% 48.3%	46.3 ± 10.0 41.0 ± 12.8	30.3% 0	3 n/a	43.2 ± 5.7 23.6 ± 3.0	21.8% n/a	?	salty	NaCl (~0.003 to ~0.034 mol/L or ~0.16 g/L to ~2 g/L)	3-AFC [62]	↑ SA
**Nance** **2017** [58]	23 8	RYGB VSG	87.0% 87.5%	43.0 ± 9.6 36.6 ± 9.9	0 0	~20% WL ~20% WL	46.9 ± 7.5 53.3 ± 8.7	19.8 ± 3.7% 19.3 ± 1.8%	12 h	sweet salty savoury	sucrose, glucose, NaCl, and MSG: (1 × 10^−4^ to 1 M)	2-AFC [61]	↔ SW, SA, SAV
**Abdeen** **2018** [48]	14 10	VSG OOC	71.4% 40.0%	15.0 ± 1.9 15.1 ± 1.8	?	3, 12 3	49.6 ± 5.9 32.0 ± 5.1	3m: 19.9 ± 1.2% 12m: 35.8 ± 1.3%	12 h	sweet	sucrose (2.1, 6.25, 12.5, 25, 50, 100, AI9300 mM)	constant stimuli [50]	↔ SW
**Nielsen** **2019** [51]	21 8 29	RYGB VSG NOC	100% 100% 100%	37.1 ± 9.9 45.0 ± 9.5 40.0 [10.4]	0 0 0	1.5, 6, 18 1.5, 6, 18 n/a	47.9 ± 6.5 43.5 ± 4.6 22.1 ± 2.4	18 m: 42.3 kg 18 m: 22.7 kg n/a	1 h	sweet	sucrose (0 g/L, 0.34 g/L, 0.55 g/L, 0.94 g/L, 1.56 g/L, 2.59 g/L, 4.32 g/L, 7.2 g/L, 12 g/L)	2-AFC [61]	↔ SW ^b^ ↑ SW ^d^ ↔ SW ^e^

Abbreviations: 2-AFC: two-alternative, forced-choice test, 3-AFC: 3-alternative forced choice test, AGB: adjusted gastric band, BI: bitter, BMI: body mass index, g: glucose, h: hour, HCl: hydrochloric acid, kg: kilogram, m: month, M: mol/L, mM: mmol/L, n/a: not applicable, NaCl: sodium chloride, NOC: non-obese control, NWC: normal weight control, SAGB: single-anastomosis gastric bypass, OOC: overweight/obese control, RYGB: Roux-en-Y gastric bypass, s: sucrose, SA: salty, SAV: savoury, SO: sour, SW: sweet, T2DM: type 2 diabetes mellitus, VLCD: very low-calorie diet, VSG: vertical sleeve gastrectomy, WL: weight loss, y: years. Data given as mean ± SD or median (interquartile range). Footnotes: ^a^ Post 1 and 2 months, but not post 3 months. ^b^ Combined both groups. ^c^ % excess weight loss. ^d^ RYGB group only. ^e^ VSG group only. ? data not reported.

**Table 3:** Key clinical parameters in the studies that investigated the effect of obesity surgery on the sensory domain of taste (recognition).

Author (year)	N	Group (s)	% Female	Age at Baseline (y)	T2DM (%)	Time after Intervention (months)	Baseline BMI (kg/m^2^)	Weight Loss (% or kg)	Time Since Meal (h)	Taste Modality	Stimuli and Concentration	Methodology	Key Results (Post vs. Pre Surgery)
**Scruggs** **1994** [52]	6 10	RYGB NWC	100% 100%	34.1 ± 7.8 36.7 ± 6.9	?	1, 2, 3 n/a	44.2 ± 7.1 20.6 ± 1.89	15, 10, 7 kg n/a	?	Sweet Sour Salty Bitter	sucrose (6–5800 mM) HCl (0.5–500 mM) NaCl (6–6100 mM) urea (90–5000 mM)	3-AFC [62]	↑ SW ^a^, SA, BI ↑↓ SO ^b^
**Burge** **1995** [11]	14 4	RYGB VLCD	57.1%	38.4 ± 6 47 ± 6	?	1.5, 3 1.5, 3	60.8 ± 11.8 43 ± 9	?	?	Sweet Bitter	sucrose (0.01- 0.1 M) urea (0.01–0.5 M)	2-AFC [61]	↑ SW ↔ BI
**Holinski** **2015** [45]	37/4/3 23	VSG/AGB/RYGB NOC	65.9% 56.2%	47.1 ± 9.8 39.5 ± 13.5	0 0	6 6	48.6 ± 7.5 23.4 ± 3.8	29.5 kg (20.6%) 0.1 kg (0.1%)	?	Sweet Sour Salty Bitter	sucrose (0.4, 0.2, 0.1, 0.05 g/mL) citric acid (0.3, 0.165, 0.09, 0.05 g/mL) NaCl (0.25, 0.1, 0.04, 0.016 g/mL) quinine HCl (0.006, 0.0024, 0.0009, 0.0004 g/mL)	Burghart taste strip [63]	↑ SW, SO, SA, BI ^e^
**Altun** **2016** [54]	52	VSG	57.7%	38.5 ± 9.4	?	1, 3	45.8 ± 7.2	1m: 25 ±7.1% ^g^ 3m: 43.9 ±10.3% ^g^	1 h	Sweet Sour Salty Bitter	sucrose (0.4, 0.2, 0.1, 0.05 g/mL) citric acid (0.3, 0.165, 0.09, 0.05 g/mL) NaCl (0.25, 0.1, 0.04, 0.016 g/mL) quinine HCl (0.006, 0.0024, 0.0009, 0.0004 g/mL)	Burghart taste strip [63]	↑ SW, SO, SA, BI
**ElLabban** **2016** [49]	9 12	RYGB VSG	33.3% 75.0%	37.0 ± 11.0 28.4 ± 7.2	?	16.8 22.8	42.8 ± 3.6 41.3 ± 4.7	38.2 kg 35.9 kg	2 h	Sweet Sour Salty Savoury	sucrose (64 mM) ^c^ citric acid 1-hydrate (8 mM) ^c^ sodium chloride (112 mM) ^c^ quinine sulfate (200 mM) ^c^	3-AFC [62]	↔ SW, SA, BI ^d^ ↓ SO ^d^
**Nielsen** **2019** [54]	21 8 29	RYGB VSG NOC	100% 100% 100%	37.1 ± 9.9 45.0 ± 9.5 40.0 [10.4]	0 0 0	1.5, 6, 18 1.5, 6, 18 n/a	47.9 ± 6.5 43.5 ± 4.6 22.1 ± 2.4	18 m: 42.3 kg 18 m: 22.7 kg n/a	1 h	Sweet	sucrose (0 g/L, 0.34 g/L, 0.55 g/L, 0.94 g/L, 1.56 g/L, 2.59 g/L, 4.32 g/L, 7.2 g/L, 12 g/L)	2-AFC [61]	↔ SW ^e^ ↑ SW ^f^

Abbreviations: BI: bitter, BMI: body mass index, DT: detection threshold, h: hour, HCl: hydrochloric acid, kg: kilogram, m: month, M: mol/L, mM: mmol/L, NaCl: sodium chloride, NOC: non obese control, RT: recognition threshold, RYGB: Roux-en-Y gastric bypass, SA: salty, SO: sour, SW: sweet, T2DM: type 2 diabetes mellitus, VLCD: very low-calorie diet, VSG: vertical sleeve gastrectomy, y: years. Data given as mean ± SD or median (interquartile range). Footnotes: ^a^ Post 1 and 2 months, but not post 3 months compared with pre-surgery. ^b^ Increased post 1 month, decreased post 2 and 3 months. ^c^ Highest levels, level 9; subsequently, eight less concentrated stimulus levels for each taste were prepared using a dilution factor of 2 of the previous level. ^d^ Comparison between RYGB vs. VSG. ^e^ Combined both groups. ^f^ 6 months after RYGB group only. ^g^ Excess weight loss. ? data not reported.

**Table 4:** Key clinical parameters in the studies that investigated the effect of obesity surgery on the sensory domain of taste (taste intensity).

Author (year)	N	Group (s)	% Female	Age at Baseline (y)	T2DM (%)	Time after Intervention (months)	Baseline BMI (kg/m^2^)	Weight Loss (% or kg)	Time Since Meal (h)	Stimuli	Stimuli and Concentration	Methodology	Key Results (Post vs. Pre Surgery)
**Pepino** **2014** [46]	17 10	RYGB/AGB	100% 100%	42.1 ± 8.4 46.8 ± 13.9	0 0	~20% WL ~20% WL	46.3 ± 7.7 48.5 ± 10.5	20.3 ± 3.0 kg 18.4 ± 2.0 kg	12 h	Sweet Sweet Salty Savoury	sucrose 0.00, 0.09, 0.36, 1.05 M glucose 0.00, 0.32, 0.56, 1.00 M NaCl 0.00, 0.056, 0.18, 0.56 M MSG 0.00, 0.02, 0.06, 0.18 M	gLMS solution random order	↓ SW (s) ^a^ ↔ SW (g), SA, SAV ^b^
**Nance** **2017** [58]	23 8	RYGB VSG	87.0% 87.5%	43.0 ± 9.6 36.6 ± 9.9	0 0	~20% WL ~20% WL	46.9 ± 7.5 53.3 ± 8.7	19.8 ± 3.7% 19.3 ± 1.8%	12 h	Sweet Sweet Salt Savoury	sucrose: 0, 90, 360, 1050 M glucose: 0, 320, 560, 1000 M NaCl: 0, 56, 180, 560 M MSG: 0, 20, 60, 180 M	gLMS solution ascending concentrations	↔ SW, SA, SAV
**Ribeiro** **2021** [53]	86 50	RYGB/VSG OC	87.5% 78%	43.5 ± 10.3 43.0 ± 9.3	16.7% 26%	12 ± 2.3 7.3 ± 4.3	42.9 ± 5.3 42.7 ± 5.0	31.9 ± 8.2% 1 ± 5.7%	1 h	Sweet Sour Salty Bitter	sucrose (0.4, 0.2, 0.1, 0.05 g/mL) citric acid (0.3, 0.165, 0.09, 0.05 g/mL) NaCl (0.25, 0.1, 0.04, 0.016 g/mL) quinine HCL (0.006, 0.0024, 0.0009, 0.0004 g/mL)	gLMS taste strip ascending concentrations	↔ SW, SO, SA, BI

Abbreviations: AGB: adjusted gastric band, BI: bitter, BMI: body mass index, gLMS: generalised labelled magnitude scale, g: glucose, HCl: hydrochloric acid, NaCl: sodium chloride, RYGB: Roux-en-Y gastric bypass, s: sucrose, SA: salty, SAV: savoury, SO: sour, SW: sweet, T2DM: type 2 diabetes mellitus, VSG: vertical sleeve gastrectomy, WL: weight loss, y: years. Data given as mean ± SD. Footnotes: ^a^ Combined both groups. ^b^ Comparison between RYGB vs. AGB.

**Table 5:** Methods used to assess reward value of a given taste.

	Method	Procedures
**Appetitive reward domain**	Progressive ratio task (PRT)	The subject must work for a rewarding stimulus; for example, this could involve clicking a computer mouse several times. The response requirement rises progressively until the subject stops making an effort for the reward, known as the breakpoint. The pioneering study of Hodos (1961) demonstrated that the number of responses made to obtain the last reward, termed the breakpoint, serves as an index of reward strength.
**Consummatory reward domain**	Category scales	Category scales are numeric and usually comprise descriptors equally spaced on a line (For example, from “1 = no taste” to “9 = extreme taste”. Common examples are the 9-point scale or the visual analogue scale (VAS).
	General labelled hedonic scale (gLHS)	The gLHS assesses pleasantness. It includes a neutral midpoint extending in opposite directions. The top anchor indicates the ‘strongest liking of any kind ever experienced’, and the bottom anchor indicates the ‘strongest disliking of any kind ever experienced’, with intermediate labels in between.
	Two series forced-choice tracking procedure	Subjects are presented with different concentration pairs of the stimulus being tested (e.g., sucrose) to identify their preference. The procedure lasts until the subject either selects a particular stimulus concentration when it is paired with a higher or lower concentration together or chooses the highest or lowest concentration two times repeatedly. The entire task is repeated with concentration pairs presented in reverse. The most preferred stimulus level is determined by the geometric mean of the concentrations chosen during the two series.
	Just about right	The participants are asked whether a sensory characteristic of the stimulus (e.g., sucrose) is too high, too low, or just about right. The scales typically comprise 5 or 7 points, ranging from too little to too much for the different stimuli.

## Data Availability

No new data were created or analysed in this study. Data sharing is not applicable to this article.
